# Analytical performance of the BD veritor™ system for rapid detection of influenza virus A and B in a primary healthcare setting

**DOI:** 10.1186/s12879-016-1811-9

**Published:** 2016-09-09

**Authors:** Sevim Mese, Hulya Akan, Selim Badur, Aysun Uyanik, Istanbul Rapid Test Study Group, Necla Icralar Emin, Dilek Dalyanci, Emine Devrim Arda, Aygul Budak, Hamit Saracoglu, Volkan Mutluer, Ece Gurgut, Emrah Kirimli, Ahmet Erdal Ugurlu, Ebru Koyuncu Dizici

**Affiliations:** 1 Department of Microbiology and Clinical Microbiology, Virology and Fundamental Immunology Unit, National Influenza Reference Laboratory, Istanbul Faculty of Medicine, Istanbul University, Istanbul, Turkey; 2 Department of Family Medicine, Faculty of Medicine, Yeditepe University, Istanbul, Turkey

**Keywords:** Seasonal influenza, Rapid diagnostic test, Point of Care, Veritor clinical sensitivity and specificity

## Abstract

**Background:**

Infections with influenza A virus cannot be clinically differentiated from infections caused by influenza B virus or other respiratory viruses. Additionally, although antiviral treatment is available for influenza A virus, it is not effective for the other viruses and must be initiated early in the course of disease for it to be effective. For these reasons, there is a need for a rapid, accurate diagnostic test for use in physicians’ offices at the time patients are seen. We report the first field performance of BD Veritor™ System for Rapid Detection of Flu A + B test compared to real time PCR. The performance of this test was compared to real time PCR performed in the Istanbul University Influenza Reference Laboratory.

**Method:**

A single-blinded cross sectional study was conducted in nine different family medicine centers in Istanbul, Turkey between 01 November 2014 and 01 May 2015. For every patient, two specimens were collected, one for real time PCR and one for the Veritor test. Specimens for the Veritor test were immediately tested at the participating clinic according to the manufacturer’s instructions. The specimens for real time PCR were transferred to the reference laboratory.

**Results:**

A total of 238 persons were included in the study: 72 (30 %) of the patients included in the study were below 19 years old and accepted as childhood group. Mean age of adults was 42.4 and children 10.2 years. A total of 122 patients out of 238 were positive for influenza. The clinical sensitivity and specificity of the Veritor test in all age groups was determined to be 80 and 94 %, respectively. Positive predictive value was 93 % and the negative one was 81 %.

**Conclusion:**

Field performance of the rapid influenza test was high and found to be useful with respect to rational antiviral use, avoiding unnecessary antibiotic usage and the management of cases by the family physicians.

## Background

Influenza is a contagious disease associated with seasonal outbreaks and significant morbidity and mortality in high risk groups [1–3]. Although the gold standard for diagnosis of influenza is virus isolation, immunoassays and real time nucleic acid amplification tests are used most commonly for laboratory diagnosis [4]. In practice, most of the cases are diagnosed clinically because in the primary healthcare services where patients most frequently present, there are no laboratory resources that can confirm the diagnosis and forwarding samples to more advanced centers causes unacceptable delays.

Clinical differentiation of influenza A virus from infections caused by influenza B virus and the other viruses is unreliable. On the other hand, effective antiviral treatment is only available for influenza A virus infections and must be administered early in the course of disease to be effective [5, 6].

Point-of-care tests are available for primary healthcare services; however, the clinical sensitivity and specificity of commercial rapid tests vary significantly. The prevalence of the disease is also important in assessing its positive and negative predictive value. During peak influenza activity, a positive test result has the highest probability of indicating the presence of infection, but a negative result does not exclude influenza infection due to low negative predictive value. In contrast, during low influenza activity such as during the warm months of the year a positive result has the lowest probability of predicting disease [7]. Rapid influenza test results are also influenced by patient age, duration of illness before specimen collection, specimen type, transport and storage conditions, and the type of influenza virus [8, 9].

Historically, rapid influenza virus antigen diagnostic tests (RIDTs) were simple to perform but were insensitive, particularly for recently introduced strains of influenza A virus and influenza B virus. In contrast, the more recently introduced digital immunoassays (DIAs) have improved analytical performance [10, 11]. The DIAs are instrument-based line immunoassays with increased clinical sensitivity and objective digital interpretation of the test results eliminating the subjectivity of reading the test results. Currently, two DIAs are FDA-approved and CLIA waived, BD Veritor System and Quidel Sofia. Although these tests are less sensitive than nucleic acid amplification tests (e.g., real time-polymerase chain reaction; RT-PCR), their performance is generally equivalent to RT-PCR early in the course of disease when virus titers are highest and antiviral treatment is most effective [12, 13]. In spite of the limitations of the tests, rapid tests can be an important option with respect to clinical decisions and disease management by the primer care [14, 15]. The use of these rapid diagnostic tests can also provide an early warning of influenza virus infections in a community [16]. For these reasons, the World Health Organization (WHO) has recomended the use of rapid tests in clinical decision making processes and early detection and control of outbreaks.

Because of most point-of-care tests are performed in primary care settings, this study was performed to assess the field performance of BD Veritor™ System for Rapid Detection of Flu A + B test compared to RT-PCR.

## Methods

This single-blinded cross sectional study was conducted in nine different family medicine centers in Istanbul, Turkey between 01 November 2014 and 01 May 2015.

### Participants

In Istanbul, there are 15 family physicians taking part in the National Influenza Sentinel Surveillance system and sending the samples to the National Influenza Laboratory of the Istanbul University. Nine family physicians participated to this study. Any patient who sought care for an influenza-like illness (ILI) and gave consent was accepted in the study. WHO criteria were used to define an ILI diagnosis [17]. Each of the physicians received training on the principles of the Veritor test, proper methods for performing the test, and the appropriate specimen collection technique.

The Public Health Institute of Turkey standardized surveillance form was completed by the family physicians for each patient and included questions regarding demographic characteristics, chronic diseases, presumed method of exposure to the respiratory virus, antiviral treatment, and the need for referral to hospital.

### Specimen collection

Nasopharyngeal specimens were collected by the physicians at the time of the intial patient visit (typically within 3 days of the onset of symptoms). Two specimens were collected, from each patient: one for RT-PCR and one for the Veritor test. Specimens for the Veritor test were immediately tested at the participating clinic according to the manufacturer’s instructions. The samples for RT-PCR were taken with a Virocult swab (Medical Wire Equipment CO, Ref No: MW 950S, England) inserted into viral transport media and stored at 4 °C for a maximum of 48 h before transport to the reference lab [18].

## BD veritor™ system for rapid detection of flu A + B

The BD Veritor™ System for Rapid Detection of Flu A + B is a rapid digital chromatographic immunoassay for the direct, qualitative detection of influenza A and B viral nucleoprotein antigens from nasal and nasopharyngeal swabs of symptomatic patients. The BD Veritor System for Rapid Detection of Flu A + B (also referred to as the BD Veritor System and BD Veritor System Flu A + B) differentiates influenza A and B viral antigens from a processed sample using a single device. Each nasopharyngeal sample was transferred to a reagent tube, mixed thoroughly, and three drops of the processed sample was dispensed into the sample well of the BD Veritor System Flu A + B test. After 10 min, the test device was inserted into the BD Veritor System reader, and test results (e.g., positive for Flu A, for Flu B, or negative) were digitally displayed by the instrument within 10 s.

### PCR testing

The swab samples were delivered to the Influenza Reference Laboratory in Istanbul University using courier system accompanied by patient information form. After checking suitability of samples and doing necessary registration, the virocult was vortexed to homogenize the specimen and then specimens were transferred to a Cryo tube (Corning cryogenic vial 2.0 ml, Ref No: CLS430659 SIGMA, England). Specimens were maintained at 4 °C if they were processed within 2 days of collection or frozen at -20 °C or -80 °C if the delay was for a longer period [18]. EZ1 Virus Mini Kit V 2.0 and Advanced XL device (QIAGEN, Ref No: 955134, Germany) was used for viral RNA extraction according to the manufacture’s instructions. The Centers for Disease Control and Prevention (CDC) RT-PCR protocol which was suggested was used. The primer-probe assays belonged to ribonucleoprotein (RNaseP) gene region, used as internal control, were provided by WHO. Amplification process was performed in the LightCycler 480 II (Roche, Germany) using Real Time ready RNA Virus Master (Light Cycler 480 RNA Master (Roche, Ref No: 05619416001, Germany) enzyme mixture. For each sample 5 μl RNA was added to a total 15 μl reaction mixture (8.6 μl molecular grade water, 0.5 μl primer, 1 μl probe, 0.4 μl enzyme and 4 μl reaction buffer). Amplification conditions were 8 min at 50 °C, 45 cycles of at 0.1 s at 95 °C + 20 s at 55 °C, + 0.1 s at 72 °C) X 45 cycle and 30 s at 40 °C. The result were reported with the analysis of amplification plot in the Light Cycler 480 II platform [19].

At the and of the study a depth interview with the physicians included in the study has been done and recorded and analyzed. The qestions were “How rapid test affected your management of upper airway infections”. The recordings were listened to, transcribed, and correlated with the notes that interviewers had taken during the interview. Each focus group transcript was read separately and during a meeting to form a coding structure. Two investigators read, identified, and assigned codes for the major themes of the data.

## Results

A total of 238 persons were included in the study: 135 women and 103 men (Table 1). A total of 30 % of the patients were less than 19 years of age with the mean age 10.2 years. The mean age of adults was 42.4 years (range 19–84). The interval between onset of symptoms and admission to family health centers was 2.2 days (range 1–7 days) in adults and was 2.2 days (range 1–8 days) in children. There was one pregnant women, one morbidly obese person and no immunosuppressive patient in the study group.

**Table 1:** Characteristics of the study group

	Childhood group *N* = 72 (%)	Adulthood group *N* = 166 (%)	Whole group *N* = 238 (%)
Female	26	109	135 (56.7 %)
Male	46	57	103 (43.3 %)
Age	10.3 ± 4.7	42.5 ± 13.7	32.7 ± 18.9
Symptom-onset interval (days)	2.8 ± 1.9	2.3 ± 1.5	2.3 ± 1.6
Seasonal Flu vaccine	2 (2.8 %)	11 (6.6 %)	13 (5.5 %)
Presence of chronic^a^ diseases	2 (2.8 %)	19 (11.4 %)	21 (8.8 %)

^a^Valid percent of chronic diseases; allergic rhinitis 4 %, asthma 1.6 %, diabetes and hepatitis C 4 %, diabetes and hypertension 4 %, epilepsy 4 %, hypothyroid 4 %, hypertension 1.2 %, hypertension and congestive cardiac failure 4 %, chronic pulmoner diseases 4 %, chronic obstructive pulmoner diseases 8 %, chronic obstructive pulmoner diseases and hypertension 4 %, breast cancer 4 %, splenectomy 4 %.

A total of 122 patients out of 238 were positive for influenza by RT-PCR including 42 with influenza A H1N1, 11 with influenza A H3N2, two with influenza A undetermined strains, and 68 with influenza B. One patient’s PCR test was positive for both influenza A and B but the Verritor test was negative for both viruses. The analytical performance of the Veritor Influenza A + B rapid test is summarized in Table 2 compared with influenza B virus infections. Overall clinical sensitivity and specificity for all patient populations was 80 and 94 %, respectively, with higher clinical sensitivity reported for patients with influenza A virus infections, (82 % versus 74 %). The differences were not statistically significant.

**Table 2:** Clinical sensitivity and specificity, predictive values of BD Veritor System for Flu AB test

	RT-PCR			
Flu rapid test	Clinical sensitivity (%)	Clinical specificity (%)	Positive predictive value (%)	Negative predictive value (%)
Influenza (A + B) in the whole study population	78 (95/122)	93 (108/116)	92 (96/104)	81 (108/134)
Influenza (A + B) in children (age < 19 years)	76 (28/37)	94 (33/35)	97 (29/31)	80 (33/41)
Influenza (A + B) in adults (age = or >19 years)	79 (67/85)	93 (75/81)	92 (67/73)	81 (75/93)
Influenza A in the whole study population (*n* = 55)	82 (45/55)	97 (177/183)	88 (45/51)	95 (177/187)
Influenza A in children (age < 19 years)	87 (14/16)	96 (54/56)	87 (14/16)	96 (54/56)
Influenza A in adult (= or >19 years)	79 (31/39)	97 (123/127)	89 (31/35)	94 (123/131)
Influenza B in the whole study population	74 (50/68)	99 (168/170)	96 (50/52)	90 (168/186)
Influenza B in children (age < 19 years)	67 (14/21)	100 (51/51)	100 (14/14)	88 (51/58)
Influenza B in adults (age = or >19 years)	77 (36/47)	97 (117/119)	95 (36/38)	91 (117/128)

Six of nine physicians attended to the interview session. The main themes that physicians have agreed are given below:

Influences on management of upper airway infections: Rapid test has increased the self confidence of physicians since faciliated the definitive diagnosis. Rapid test has also inceased their awareness of that the flu season has begun. Also they expressed that it has also increased the confidence of patients and had positive effect on doctor-patient relationship. It also increased the examination time due to the physicians spend more time for consultation of preventive measures to be taken and acknowledgement of about the disease. It had not specific effect on reference of secondary-tertiary care.

Influences on prescription: In general rapid test has not changed the prescriptions. In few patients physicians expressed that they had oppurtunity to convince patients to not to use antibiotics. Also, it has faciliated to decide prescribing antivirals.

In general since PCR test results have arrived in one or two days, a tool supporting the immediate diagnosis has positively effected the management and follow-up of the patients admitted with flu symptoms. The overall feedback from the family physicians was positive during the physician group interviews after the study. Although the family physicians expressed that their prescriptions were not significantly influenced by the test results, the test facilitated the decision to prescribe or not to prescribe antibiotics and was also useful in reasurring their patients not to use antibiotics. In general, the greatest benefit of the test was useful for explaining the disease and routes of contamination to the patients, and the risks of potential complications associated with influenza virus infections.

## Discussion

The historical gold standard for detection of influenza virus is virus isolation in cell culture. In practice, virus isolation has been replaced by RT-PCR in most reference laboratories [20]; however, RT-PCR tests are not performed in the primary healthcare setting because of the technical complexity of these tests. Additionally, because of the delays associated with transport of specimens to reference labs and the time required for performance of RT-PCR tests and receipt of results from reference laboratories is typically too slow to be useful for the clinical management of patients [21]. This delay usually has a negative impact on appropriate antiviral treatment and prevention of transmission, particularly in individuals living with others who are in high risk groups [6, 22, 23]. A rapid accurate test that can be performed in the primary care setting would facilitate the management of suspected influenza virus infections.

Rapid influenza diagnostic tests (RIDTs) have historically been too insensitive to be used reliably for patient care; however, the newly introduced DIAs are reported to have superior performance [10, 11, 24, 25]. These studies have been performed in well-controlled, centralized laboratories or during clinical trials, so the performance may not reflect the results obtained in busy primary care facilities. For that reason, we performed this multisite study. In this study in a primary care setting, the BD Veritor System Flu A + B test had excellent clinical sensitivity and specificity in all age groups (80 and 94 %, respectively) and the positive and negative predictive values were 93 and 81 %, respectively, in a population with a 51 % prevalence of disease.

In a study comparing three different rapid antigen tests, BD Veritor System Influenza A + B test was found to have a good performance. In a study conducted in children under 18 years of age, positive predictive value was found to be 91.8 % and negative predictive value was 98.4 % [25]. In another study comparing three different rapid antigen tests, it was concluded that the Veritor Influenza A + B test was superior to the other tests [26].

In the influenza season of the 2014–2015 when the study was conducted, initial cases were seen in January and the seasonal outbreak began in February and continued until the middle of April. We believe the incidence of influenza infections in the study population reflected disease in the general population. In The National Influenza Reference Laboratory of Istanbul University reported 55.1 % of 2068 patients were infected with influenza B, 35.4 % with influenza A H1N1, and 9.4 % with influenza A H3N2, similar to the distribution observed in this study. It is known that the positive and negative predictive values of diagnostics are affected by the prevalence of disease [7]. In this study, the positive predictive values for influenza B was higher (95 % versus 100 %) and the negative predictive values for influenza A was higher (94 % versus 96 %). The test detected most of the influenza B cases immediately. This result can be explained by the fact that cases in seasonal outbreaks are predominantly influenza B cases.

Rapid tests reduce additional test requests in both primary healthcare and emergency services, increase the rate of rational antiviral treatment, decrease the rate of inappropriate antibiotic use and impact clinical decision making in general in a positive manner [27–30]. The goal of this study was to assess the performance of the rapid Veritor Flu A + B test. Because performance in a primary care facility was unknown, no effort was made to measure the influence of the test result on patient management. Clearly this would be an important future study.

However, the overall feedback from the family physicians was positive during the physician group interviews after the study was completed. The family physicians test results, the test facilitated the decision antiviral and useful in reassuring the patients not to use antibiotics when a negative result was obtained. Additionally the physicians felt test was useful for initiating a discussion about influenza virus infections, routes of exposure and transmission, and the risks of potential complications associated with influenza virus infections.

The result of this study were potentially limited because it was conducted in a single season and performance was based on the circulating strains of influenza A and B viruses during this season. Results may be different in a seasonal outbreak when other strains of influenza predominate although this is unlikely because the assay target is a stable ribonucleoprotein, and not the neuraminidase and hemagglutinin antigens that are responsible for the strain heterogeneity. Although the time between the onset of the symptoms and performance of the test can influence the results, most tests were performed within 4 days of onset of symptoms which is reflective of an out-patient practice. The number of patients in the high risk population of less than 5 years of age was low in this study, but the clinical sensitivity and specificity for these patients was 100 %. Test results can also be influenced by the method of specimen collection or compliance with testing methods, but we believe the performance of this Clinical Laboratory Improvement Amendments-waived test is reflective of common practice in out-patient settings.

## Conclusions

In conclusion, the field performance of the Veritor Flu A + B test was high for both pediatric and adult patients when performed in a primary care setting. Additional studies to demonstrate the clinical and financial impact of this assay are warranted.

## Abbreviations

CDC: Centers for Disease Control and Prevention
DIAs: Digital immunoassays
RIDTs: Rapid influenza virus antigen diagnostic tests
RnaseP: Ribonucleoprotein
RT-PCR: Real time-polymerase chain reaction
WHO: World Health Organization
